# Supplementary figures and images for: Presence of Pseudomonas aeruginosa in feces exacerbate leaky gut in mice with low dose dextran sulfate solution, impacts of specific bacteria
Source: PLoS One. 2024 Nov 15;19(11):e0309106. doi: 10.1371/journal.pone.0309106 (PMC11567622; doi:10.1371/journal.pone.0309106)

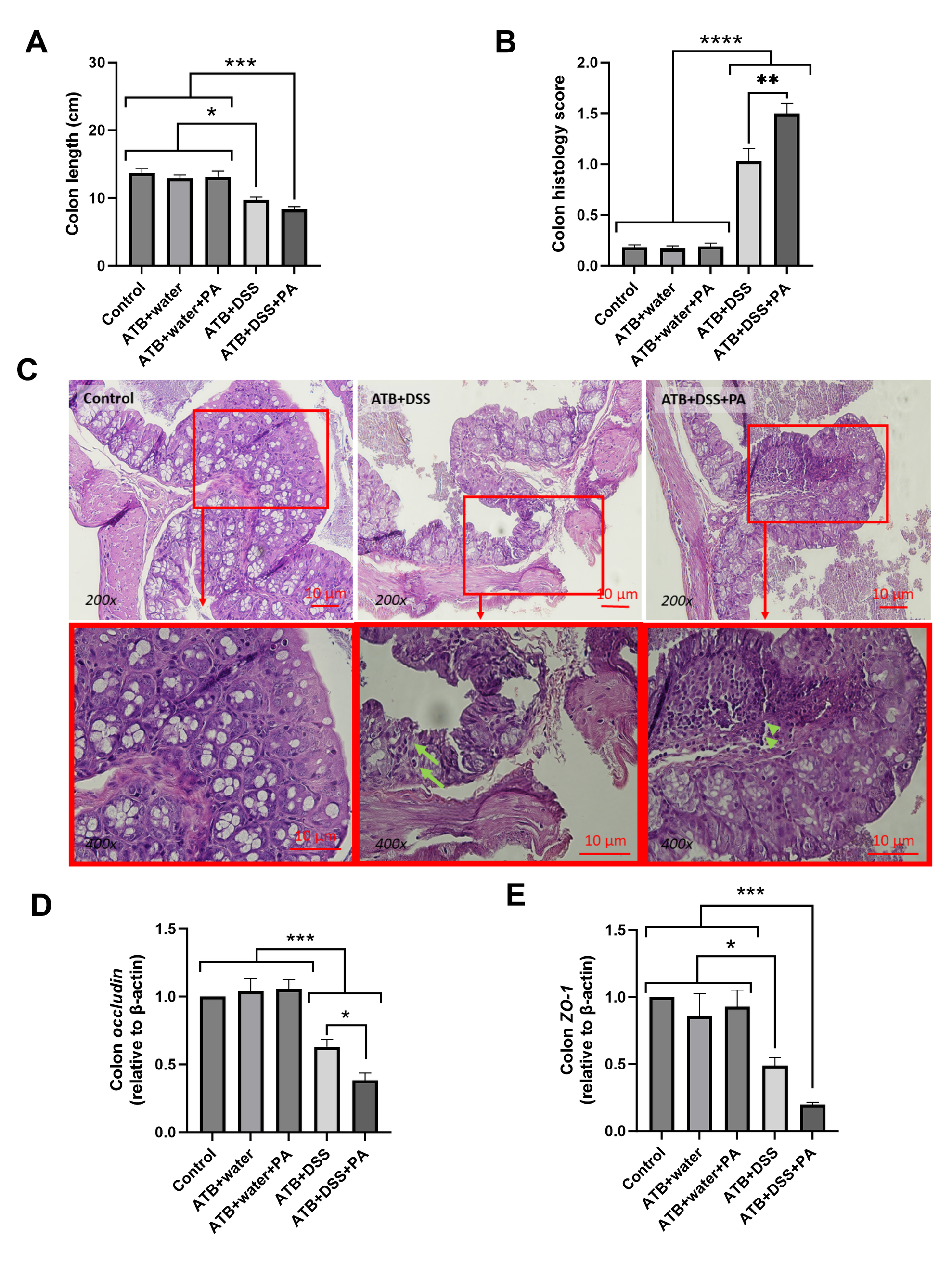

Supplement: S1 Fig — Characteristics of mice in the non-DSS groups, including water control (control), antibiotics without or with P. aeruginosa (ATB+water and ATB+water+PA), and the DSS groups, including dextran sulfate solution (DSS) without or with P. aeruginosa (ATB+DSS and ATB+DSS+PA) as indicated by colon length (A), intestinal injury score with the representative histopathology (Hematoxylin and eosin stain) (B, C). and expression of tight junction molecules (occludin-1 and ZO-1) (D) are demonstrated (n = 5–7/group). Significant differences *, p < 0.05; **, p < 0.01; ***, p < 0.001; ****, p < 0.0001 were compared between the indicated groups. The representative intestinal pictures of ATB+water and ATB+water+PA are not demonstrated due to the similarity to the control group. Arrow; inflammatory cells, arrow head; inflammatory cells in group. (TIF) [file pone.0309106.s001.tif]
