# Supplementary material for: Presence of Pseudomonas aeruginosa in feces exacerbate leaky gut in mice with low dose dextran sulfate solution, impacts of specific bacteria
Source: PLoS One. 2024 Nov 15;19(11):e0309106. doi: 10.1371/journal.pone.0309106 (PMC11567622; doi:10.1371/journal.pone.0309106)
Supplement: Attachment — Submitted filename: Response to Reviewers.docx [file pone.0309106.s002.docx]

PONE-D-24-13045

Presence of Pseudomonas aeruginosa in feces exacerbate leaky gut in mice with low dose dextran sulfate solution, impacts of specific bacteria

PLOS ONE

Dear Dr. Leelahavanichkul,

Thank you for submitting your manuscript to PLOS ONE. After careful consideration, we feel that it has merit but does not fully meet PLOS ONE’s publication criteria as it currently stands. Therefore, we invite you to submit a revised version of the manuscript that addresses the points raised during the review process.

It was felt that the authors need to provide the data pertaining to at least the expression of genes for the tight junction proteins.  In addition, a rationale for the use of 2h time point for the effect on enterocytes, whereas 4h time point used for the gene expression profile for the hepatocytes should be provided.  Also, several details including the type of LPS was missing.  Please submit the responses to each of the reviewer's concerns.

ANS: We thank the editor for the comment and we provide the tight junction proteins gene expression in colon of each group of mice. Moreover, we add the discussion on the result section about a rationale for the use of 2h time point for the effect on enterocytes, whereas 4h time point used for the gene expression profile for the hepatocytes at the lines 443-445 and 467-473.

Please submit your revised manuscript by Jun 29 2024 11:59PM. If you will need more time than this to complete your revisions, please reply to this message or contact the journal office at <a ref="mailto:plosone@plos.org">plosone@plos.org. When you're ready to submit your revision, log on to https://www.editorialmanager.com/pone/ and select the 'Submissions Needing Revision' folder to locate your manuscript file.

Please include the following items when submitting your revised manuscript:

- A rebuttal letter that responds to each point raised by the academic editor and reviewer(s). You should upload this letter as a separate file labeled 'Response to Reviewers'.
- A marked-up copy of your manuscript that highlights changes made to the original version. You should upload this as a separate file labeled 'Revised Manuscript with Track Changes'.
- An unmarked version of your revised paper without tracked changes. You should upload this as a separate file labeled 'Manuscript'.

If you would like to make changes to your financial disclosure, please include your updated statement in your cover letter. Guidelines for resubmitting your figure files are available below the reviewer comments at the end of this letter.

If applicable, we recommend that you deposit your laboratory protocols in protocols.io to enhance the reproducibility of your results. Protocols.io assigns your protocol its own identifier (DOI) so that it can be cited independently in the future. For instructions see: <https://journals.plos.org/plosone/s/submission-guidelines#loc-laboratory-protocols>. Additionally, PLOS ONE offers an option for publishing peer-reviewed Lab Protocol articles, which describe protocols hosted on protocols.io. Read more information on sharing protocols at <https://plos.org/protocols?utm_medium=editorial-email&utm_source=authorletters&utm_campaign=protocols>.

We look forward to receiving your revised manuscript.

Kind regards,

Pradeep Dudeja
Academic Editor
PLOS ONE

Journal Requirements:

When submitting your revision, we need you to address these additional requirements.

1. Please ensure that your manuscript meets PLOS ONE's style requirements, including those for file naming. The PLOS ONE style templates can be found at 
https://journals.plos.org/plosone/s/file?id=wjVg/PLOSOne_formatting_sample_main_body.pdf and 
https://journals.plos.org/plosone/s/file?id=ba62/PLOSOne_formatting_sample_title_authors_affiliations.pdf

ANS: We thank the editor for the comment and we ensure that our manuscript meets PLOS one’ s style requirements.

2. Please note that funding information should not appear in any section or other areas of your manuscript. We will only publish funding information present in the Funding Statement section of the online submission form. Please remove any funding-related text from the manuscript.

ANS: We remove funding from the revised manuscript.

3. Thank you for stating the following financial disclosure: 
   "This research is supported by the Program Management Unit for Human Resources & Institutional Development, Research and Innovation (B16F640175) with Rachadapisek Sompote Matching Fund (RA-MF-22/65 and RA-MF-13/66), and Rachadapisek Sompote Endowment Fund (RA66/008 and RA66/009), as well as National Research Council of Thailand (NRCT-N41A640076 and NRCT-N34A660583). WP was supported by Rachadapisek Sompote Fund for Postdoctoral Fellowship, Chulalongkorn University. "

Please state what role the funders took in the study.  If the funders had no role, please state: "The funders had no role in study design, data collection and analysis, decision to publish, or preparation of the manuscript." 
If this statement is not correct you must amend it as needed. 
Please include this amended Role of Funder statement in your cover letter; we will change the online submission form on your behalf.

ANS: We amend the role of funders “The funders had no role in study design, data collection and analysis, decision to publish, or preparation of the manuscript."

4. We note that your Data Availability Statement is currently as follows: All relevant data are within the manuscript and its Supporting Information files.

Please confirm at this time whether or not your submission contains all raw data required to replicate the results of your study. Authors must share the “minimal data set” for their submission. PLOS defines the minimal data set to consist of the data required to replicate all study findings reported in the article, as well as related metadata and methods (https://journals.plos.org/plosone/s/data-availability#loc-minimal-data-set-definition).

For example, authors should submit the following data:

- The values behind the means, standard deviations and other measures reported;
- The values used to build graphs;
- The points extracted from images for analysis.

ANS: We thank the editor for the comment and we provide the minimal data set for publication in the excel file.

Authors do not need to submit their entire data set if only a portion of the data was used in the reported study.

If your submission does not contain these data, please either upload them as Supporting Information files or deposit them to a stable, public repository and provide us with the relevant URLs, DOIs, or accession numbers. For a list of recommended repositories, please see https://journals.plos.org/plosone/s/recommended-repositories.

If there are ethical or legal restrictions on sharing a de-identified data set, please explain them in detail (e.g., data contain potentially sensitive information, data are owned by a third-party organization, etc.) and who has imposed them (e.g., an ethics committee). Please also provide contact information for a data access committee, ethics committee, or other institutional body to which data requests may be sent. If data are owned by a third party, please indicate how others may request data access.

5. When completing the data availability statement of the submission form, you indicated that you will make your data available on acceptance. We strongly recommend all authors decide on a data sharing plan before acceptance, as the process can be lengthy and hold up publication timelines. Please note that, though access restrictions are acceptable now, your entire data will need to be made freely accessible if your manuscript is accepted for publication. This policy applies to all data except where public deposition would breach compliance with the protocol approved by your research ethics board. If you are unable to adhere to our open data policy, please kindly revise your statement to explain your reasoning and we will seek the editor's input on an exemption. Please be assured that, once you have provided your new statement, the assessment of your exemption will not hold up the peer review process.

ANS: All data were included in the minimal data set for publication. If you require additional information, please contact the corresponding author.

[Note: HTML markup is below. Please do not edit.]

Reviewers' comments:

Reviewer's Responses to Questions

**Comments to the Author**

1. Is the manuscript technically sound, and do the data support the conclusions?

The manuscript must describe a technically sound piece of scientific research with data that supports the conclusions. Experiments must have been conducted rigorously, with appropriate controls, replication, and sample sizes. The conclusions must be drawn appropriately based on the data presented.

Reviewer #1: Yes

Reviewer #2: Yes

2. Has the statistical analysis been performed appropriately and rigorously?

Reviewer #1: No

Reviewer #2: Yes

3. Have the authors made all data underlying the findings in their manuscript fully available?

The [PLOS Data policy](http://www.plosone.org/static/policies.action#sharing) requires authors to make all data underlying the findings described in their manuscript fully available without restriction, with rare exception (please refer to the Data Availability Statement in the manuscript PDF file). The data should be provided as part of the manuscript or its supporting information, or deposited to a public repository. For example, in addition to summary statistics, the data points behind means, medians and variance measures should be available. If there are restrictions on publicly sharing data—e.g. participant privacy or use of data from a third party—those must be specified.

Reviewer #1: Yes

Reviewer #2: Yes

4. Is the manuscript presented in an intelligible fashion and written in standard English?

PLOS ONE does not copyedit accepted manuscripts, so the language in submitted articles must be clear, correct, and unambiguous. Any typographical or grammatical errors should be corrected at revision, so please note any specific errors here.

Reviewer #1: Yes

Reviewer #2: Yes

5. Review Comments to the Author

Please use the space provided to explain your answers to the questions above. You may also include additional comments for the author, including concerns about dual publication, research ethics, or publication ethics. (Please upload your review as an attachment if it exceeds 20,000 characters)

Reviewer #1: This study demonstrates that the presence of pseudomonas aeruginosa (PA) in the gut exacerbated DSS-induced intestinal injury with spontaneous translocation of LPS and bacteria from the gut into the blood circulation that induced severe systemic inflammation using mice as an in vivo model system. Although the study is interesting, there are several issues that need to be addressed.

1. Statistical analysis must be done for the entire data presented in this manuscript. Most of the p-values presented appears to be wrong.

ANS: We thank the reviewer for the comment and we check, correct all statistical analysis in all figures. The p-values have been changed.

2. Provide colon length and morphology of controls and ATB+PA+DSS treated mice.

ANS: We thank the reviewer for the comment and we provide colon length and morphology of controls and ATB+PA+DSS treated mice in supplement data (S1 Fig).

3. Specify the type of LPS used in this study.

ANS: We thank the reviewer for the comment and we add the type of LPS in revised manuscript in the method section “lipopolysaccharide (LPS) from *Escherichia coli* O26:B6 (Sigma-Aldrich) at 1 µg/mL” at the lines 263-264.

4. Provide the tight junction proteins gene expression levels in controls and ATB+PA+DSS treated mice colon.

ANS: We thank the reviewer for the comment and we provide the tight junction proteins gene expression level in controls and ATB+PA+DSS treated mice colon in the supplement data at the lines 310-311.

5. Many typographical and grammatical errors throughout the manuscript.

ANS: We thank the reviewer for the comment and we check and correct the typographical and grammatical errors throughout the revised manuscript and labeled with yellow highlight.

Reviewer #2: In this interesting study, the authors tested the hypothesis that increased abundance of pathogenic bacteria (specifically of the phylum Proteobacteria) in healthy hosts may increase of the healthy hosts their susceptibility to intestinal injury. The authors investigated the impact of Pseudomonas aeruginosa (PA, the representative Proteobacteria) in a mouse model with non-diarrheal gut permeability defect using 1.5% dextran sulfate solution (DSS) plus antibiotics (ATB) with or without orally administered PA. The results showed that ATB+DSS+PA induced more severe intestinal injury as indicated by stool consistency and leaky gut (FITC dextran assay, bacteremia, and endotoxemia) with an increase in serum cytokines, liver enzyme, and hepatocyte apoptosis when compared with ATB+DSS mice. However, these parameters remained unchanged in the non-DSS group: water alone (Control), antibiotics alone (ATB+water), and antibiotics with PA (ATB+water+PA). A similar fecal microbiome pattern was observed between ATB+DSS and ATB+DSS+PA. Moreover, a higher abundance of Pseudomonas, Enterococci, and Escherichia-Shigella was detected in ATB+DSS+PA compared to ATB+DSS. Additionally, heat-killed P. aeruginosa (PA) and LPS together induced enterocyte (Caco2 cells) injury and hepatic injury compared to LPS or PA alone via increased expression of proinflammatory markers (TNF-α, IL-8, and NOS2) and apoptosis proteins (caspase3, caspase8, Bcl2). Based on these data, the authors concluded that the presence of P. aeruginosa in the gut of mice with DSS-induced non-diarrheal mucosal injury exacerbated prominent leaky gut that induced more severe systemic inflammation.
This study has clinical relevance as it can provide insights on how to interpret and further manage healthy individuals with a high fecal abundance of pathogenic bacteria, especially Pseudomonas spp., Klebsiella spp., and Salmonella spp. that still remains a challenge. The abstract is concise, and the conclusions are well supported by the relevant data. However, the authors should consider the following suggestions:

1. Did the authors examine the expression level of tight junction proteins such as occludin, claudins 1, 2, 4 and ZO1 in ATB+DSS+PA mice compared to ATB+DSS mice?

The authors should provide a compelling rationale as to why both mice were used for the current study.

ANS: We thank the reviewer for the comment and we provide the tight junction proteins gene expression in colon of each group of mice at the lines 310-311 (S1 Fig).

We add the rationale why both mice were used for this study in the Fig. 9 and in conclusion section.

2. Any specific reason as to why 2h (enterocytes) and 4h (hepatocytes) time points were chosen to examine the effects of PA + LPS on the expression various genes including IL8, TNF-α, NOS2, caspases 3, 8, 9 and Bcl2?

ANS: We thank the reviewer for the comment and we answer the specific reason in this point.

Because peak of the cytokine production from enterocytes was at 24 h after incubation, the expression of the cytokine genes might be earlier and was performed at 2 h post-incubation [30, 34] at the lines 443-445.

While the peak of cytokine production from the stimulated enterocytic cells was at 24 h post-incubation (Fig 7A), hepatocytic cytokine production peaked approximately at 72 h (Figs 8A-C). Then, gene expression of cytokines in enterocytes and hepatocytes was performed at 2 and 4 h, respectively, as described in a previous publication [30]. Hence, the gene expression of other mechanisms that might be correlated with cytokines, including inflammatory process (*NOS2*), and apoptosis (*casp3*, *casp8*, *casp9* and Bcl2) was also performed at 4 h at the lines 467-473.

3. Figure legends 1-3 (lines 70, 709, 717)- should be ATB + water + PA instead of ATB + ATB + PA

ANS: We thank the reviewer for the comment and we correct accordingly.

4. Figure legend 7 (line 741)- should be 2h instead of 4h

ANS: We thank the reviewer for the comment and we correct accordingly.

5. Line 288- It should be colon not ileum

ANS: We thank the reviewer for the comment and we correct accordingly.

6. The authors should proof-read the manuscript carefully to avoid typo and grammatical errors.

ANS: We thank the reviewer for the comment and we thoroughly proof-read all sentences in revised manuscript.

6. PLOS authors have the option to publish the peer review history of their article ([what does this mean?](https://journals.plos.org/plosone/s/editorial-and-peer-review-process#loc-peer-review-history)). If published, this will include your full peer review and any attached files.

If you choose “no”, your identity will remain anonymous but your review may still be made public.

**Do you want your identity to be public for this peer review?** For information about this choice, including consent withdrawal, please see our [Privacy Policy](https://www.plos.org/privacy-policy).

Reviewer #1: No

Reviewer #2: No

[NOTE: If reviewer comments were submitted as an attachment file, they will be attached to this email and accessible via the submission site. Please log into your account, locate the manuscript record, and check for the action link "View Attachments". If this link does not appear, there are no attachment files.]

While revising your submission, please upload your figure files to the Preflight Analysis and Conversion Engine (PACE) digital diagnostic tool, <https://pacev2.apexcovantage.com/>. PACE helps ensure that figures meet PLOS requirements. To use PACE, you must first register as a user. Registration is free. Then, login and navigate to the UPLOAD tab, where you will find detailed instructions on how to use the tool. If you encounter any issues or have any questions when using PACE, please email PLOS at <a href="mailto:figures@plos.org">figures@plos.org. Please note that Supporting Information files do not need this step.
